# Persistent Lupus Anticoagulant Positivity and Long-Term Sequelae Following Mild COVID-19

**DOI:** 10.7759/cureus.72668

**Published:** 2024-10-29

**Authors:** Yasutaka Kuniyoshi

**Affiliations:** 1 Department of Social Services and Healthcare Management, International University of Health and Welfare, Otawara, JPN

**Keywords:** activated partial thromboplastin time (aptt), antiphospholipid antibody, covid-19, lupus anti-coagulant, post-covid-19 conditions (pcc)

## Abstract

We report a case of persistent lupus anticoagulant (LAC) positivity following mild COVID-19 in a 64-year-old Japanese male with a history of atrial fibrillation. The patient experienced post-COVID-19 condition symptoms, including intermittent fatigue, taste disturbance, and persistent numbness in the upper arm, persistently for over 10 months. Laboratory investigations revealed prolonged activated partial thromboplastin time (aPTT) of 70.0 seconds, positive LAC of 1.5, and positive anti-cardiolipin-beta2-glycoprotein I complex antibody of 4.3 U/mL. This case highlights the potential for long-term LAC positivity after mild COVID-19 and raises questions about its association with post-COVID-19 conditions. The persistence of LAC positivity is noteworthy, as previous studies suggest that virus-induced LAC typically resolves within two to three months. Further research is needed to elucidate the long-term dynamics of LAC in post-COVID-19 condition patients and its clinical implications, particularly in relation to thrombotic complications associated with COVID-19.

## Introduction

Coronavirus disease 2019 (COVID-19), caused by the severe acute respiratory syndrome coronavirus 2 (SARS-CoV-2), is associated with a wide range of clinical manifestations, including thrombotic events. Both venous and arterial thrombosis can occur, which is unusual compared to other respiratory infections [1]. Venous thromboembolism is a common complication in COVID-19 patients, with studies reporting incidence rates three to six times higher than in patients hospitalized for other reasons [2]. Recent studies have reported an increased prevalence of lupus anticoagulant (LAC) positivity in patients with COVID-19 during the acute phase, approximately 50% [3,4]. While the association between COVID-19 severity and LAC has been investigated, the long-term persistence of LAC positivity and its potential implications for post-COVID-19 conditions remain unclear. This case report presents a patient who developed persistent LAC positivity following mild COVID-19 and experienced long-term sequelae.

## Case presentation

A 64-year-old Japanese male presented to our clinic in May 2024 for a second opinion regarding persistent prolongation of the activated partial thromboplastin time (aPTT) following SARS-CoV-2 infection. The patient had a past medical history of atrial fibrillation, for which he underwent catheter ablation five years prior. He had since maintained a stable sinus rhythm. He had been prescribed long-term oral anticoagulation with apixaban 2.5 mg twice daily by his attending cardiologist. He also had hyperlipidemia managed with pitavastatin 1mg daily. The patient was a non-smoker and reported no alcohol consumption. He worked as a university faculty member. The patient had received five doses of the COVID-19 mRNA vaccine: four doses of the Pfizer-BioNTech (Pfizer, USA; BioNTech, Germany) vaccine and one dose of the Moderna vaccine (Moderna, Inc., USA), with the last dose administered in November 2022.

In July 2023, the patient was diagnosed with COVID-19, confirmed by SARS-CoV-2 antigen rapid testing. He experienced mild symptoms including fever and cough and was treated with supportive care without antiviral agents. He did not require hospitalization or supplemental oxygen therapy. Following the acute infection, intermittent fatigue, taste disturbance, and numbness in the upper arm persisted for more than 10 months, consistent with the post-COVID-19 condition. He had no clinical findings associated with antiphospholipid antibody syndrome.

On presentation in May 2024, his vital signs were temperature 36.3°C, pulse 72 beats per minute, blood pressure 122/79 mmHg, and oxygen saturation 98% on room air. Physical examination revealed no significant abnormalities in the cardiovascular, respiratory, or abdominal systems.

**Figure 1:**
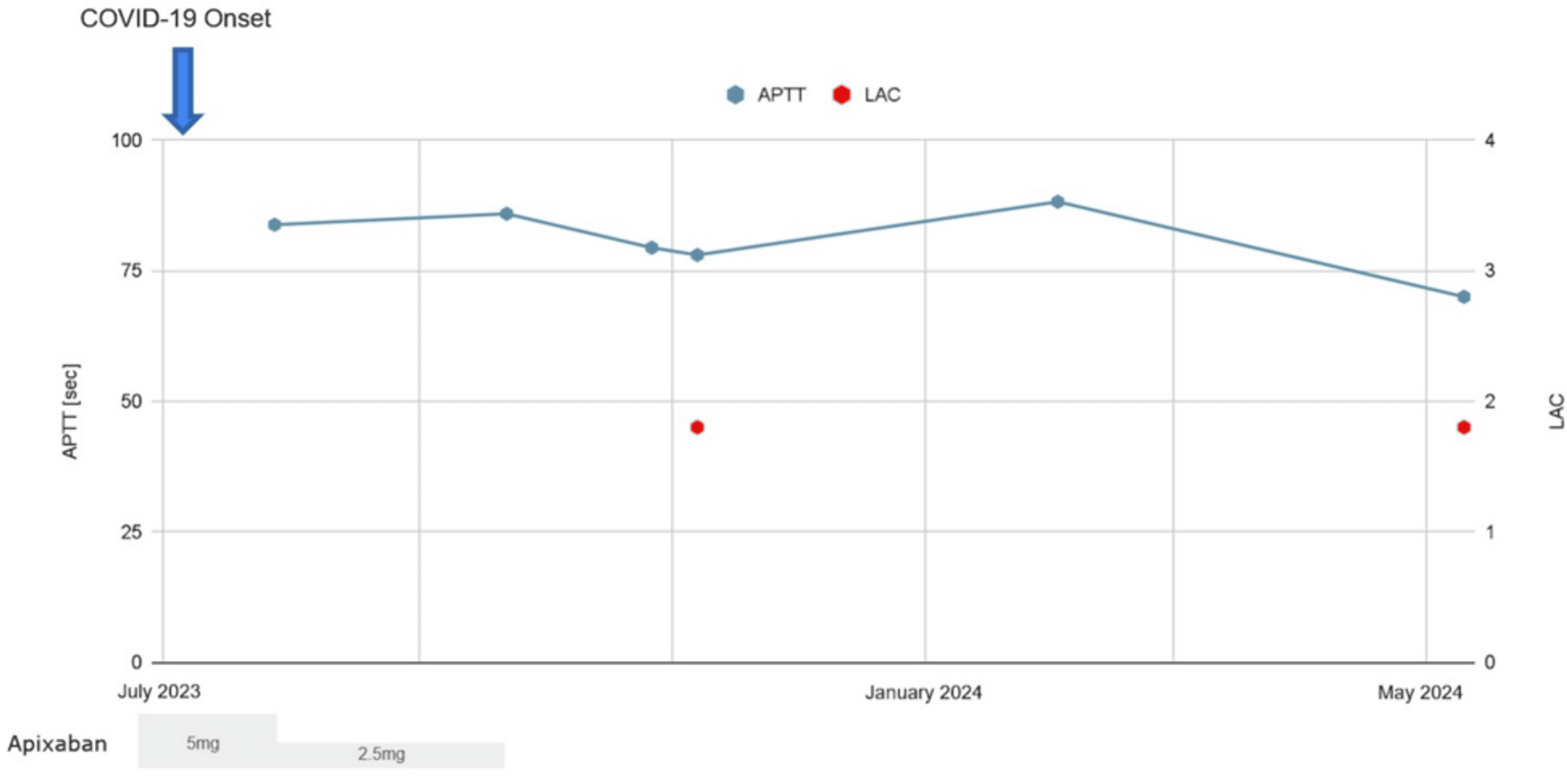
Trends in activated partial thromboplastin time and lupus anticoagulant during after COVID-19 onset APTT: activated partial thromboplastin time, LAC: lupus anticoagulant

With oral anticoagulants discontinued, 10 months after the initial COVID-19, laboratory investigations revealed prolonged aPTT of 70.0 seconds (reference range: 24-39 seconds), positive LAC of 1.5 (reference range: ≤1.2), and positive anti-cardiolipin-beta2-glycoprotein I complex antibody (CLβ2-GP1) of 4.3 U/mL (reference range: <3.5 U/mL). Other blood tests, including complete blood count and biochemical parameters, were within normal limits (Table 1). Figure 1 illustrates the trend of aPTT and LAC over time, showing persistent elevation following COVID-19.

**Table 1:** Laboratory test results at 10 months onset ANA: antinuclear antibody, anti-dsDNA: anti-double-stranded DNA IgG antibody, LAC: lupus anticoagulant, dRVVT: dilute Russell's viper venom time, CLβ2-GP1: anti-cardiolipin-beta2-glycoprotein I complex antibody

	Laboratory values	Units
WBC	4,500	/μL
RBC	474	×10^4^/μL
Hb	14.8	g/dL
Ht	43.8	%
PLT	21.1	×10^4^/μL
%PT	95	%
APTT	70.0	sec
Fib	278	mg/dL
D-dimer	<0.5	μg/mL
AST	20	U/L
ALT	24	U/L
LDH	160	U/L
CPK	65	U/L
TP	6.9	g/dL
ALB	4.3	g/dL
BUN	14	mg/dL
CRN	0.91	mg/dL
C3	89	mg/dL
C4	17	mg/dL
IgG	1126 (reference, 870-1,700)	mg/dL
IgM	90 (reference, 33-190)	mg/dL
ANA	40	
anti-dsDNA	<10 (reference: ≦12)	IU/mL
LAC	1.5 (reference: ≦1.2)	
CLβ2-GP1	4.3 (reference: <3.5)	U/mL

## Discussion

This case report highlights the potential for long-term LAC positivity following mild COVID-19. It is important to note that further prolongation of aPTT in patients on anticoagulation therapy after COVID-19 does not necessarily require discontinuation of anticoagulation therapy. Instead, it should prompt confirmation of LAC presence and careful monitoring.

The exact mechanisms underlying the development of LAC following COVID-19 are not fully understood. Several hypotheses have been proposed, including direct viral infection of endothelial cells [5,6], hypercoagulable state [7], and consequence of the acute inflammatory state [8,9]. However, the relationship between LAC positivity and clinical outcomes in COVID-19 patients remains controversial. Some studies have not found a significant association between LAC positivity alone and increased risk of venous thromboembolism or in-hospital mortality in COVID-19 patients [10]. Further research is needed to elucidate the pathogenesis and clinical significance of LAC positivity in the context of COVID-19.

The duration of LAC positivity following the acute phase of COVID-19 remains unclear. A study reported that LAC positivity observed during the acute phase may resolve within a few months, while other antiphospholipid antibodies such as CLβ2-GP1 may persist [11]. In our patient, aPTT remained elevated for an extended period after the acute phase, with confirmed LAC positivity 10 months post-infection. This persistence of LAC positivity is noteworthy, as previous studies have suggested that virus-induced LAC other than SARS-CoV-2 typically becomes negative within two to three months [12].

The persistence of LAC positivity in this case, along with the patient's experience of long-term sequelae, raises questions about potential associations between persistent LAC positivity and post-COVID-19 conditions. Although several studies have identified potential biomarkers characteristic of patients with post-COVID-19 conditions, there is no single definitive test marker to diagnose post-COVID-19 conditions [13]. This hypothesis has not been extensively tested in previous studies and warrants further investigation.

A limitation of this case report is that we cannot completely rule out the possibility that LAC was positive before the onset of COVID-19. However, the temporal relationship between COVID-19 and aPTT prolongation supports the hypothesis that SARS-CoV-2 infection induced LAC positivity in this patient.

## Conclusions

This case report describes a patient who developed persistent LAC positivity and experienced long-term sequelae following mild COVID-19. As a definitive diagnosis for post-COVID-19 conditions is not yet established, the persistence of LAC positivity and its potential association with post-COVID-19 condition warrant further investigation. Future studies should aim to elucidate the long-term dynamics of LAC in post-COVID-19 condition patients and its clinical implications.
